# Treatment for retropharyngeal metastatic undifferentiated squamous cell carcinoma from an unknown primary site: results of a prospective study with irradiation to nasopharyngeal mucosa plus bilateral neck

**DOI:** 10.18632/oncotarget.16344

**Published:** 2017-03-18

**Authors:** Chengrun Du, Hongmei Ying, Youwang Zhang, Yafang Huang, Ruiping Zhai, Chaosu Hu

**Affiliations:** ^1^ Department of Radiation Oncology, Fudan University Shanghai Cancer Center, Shanghai, P.R. China; ^2^ Department of Ultrasound, Fudan University Shanghai Cancer Center, Shanghai, P.R. China

**Keywords:** retropharyngeal nodes, undifferentiated squamous cell carcinoma, transoral sonography-guided fine-needle aspiration, primary unknown, radiotherapy

## Abstract

**Background and Objective:**

To evaluate treatment outcomes for patients with retropharyngeal metastatic undifferentiated squamous cell carcinoma (SCC) from an unknown primary site.

**Methods:**

From January 2005 to January 2015, patients who presented with enlarged retropharyngeal nodes underwent transoral sonography-guided fine-needle aspiration to confirm histology. Those with metastatic undifferentiated SCC with unknown primary tumors were treated with radical radiotherapy to nasopharyngeal mucosa plus bilateral neck. Chemotherapy was administered for patients staged N2-3. Endpoints included metastatic nodes control, the appearance of primary tumor, overall survival and treatment-related toxicities.

**Results:**

A total of 49 patients were recruited into this study. Retropharyngeal and cervical nodal disease was controlled in 96% of all patients. The incidence of occult primary cancer appearance was 8%. No primary cancer other than of the nasopharynx was detected during the course of follow-up. Ten patients developed distant metastases. The 5-year overall survival, progression-free survival, regional relapse free survival, distant metastasis free survival were 79.6%, 61.1%, 83.4%, 73.8%, respectively. Common late adverse effects included xerostomia (57%) and hearing impairment (35%).

**Conclusion:**

Radical radiotherapy to both the nasopharynx and bilateral neck can achieve excellent outcome with mild toxicities for patients with retropharyngeal metastatic undifferentiated squamous cell carcinoma from an unknown primary site.

## INTRODUCTION

The retropharyngeal lymph nodes (RLNs) are known as Rouviere nodes, which are located medially to the internal carotid artery. Enlarged retropharyngeal nodes can be the result of inflammatory diseases or malignancy. Pathologic types of metastatic carcinoma in retropharyngeal nodes can be various. It's reported that carcinoma of head and neck, thyroid cancer and even esophagus cancer had the ability to metastasize to retropharyngeal nodes [1–3].

Retropharyngeal lymph nodes are usually considered as the first-echelon lymph nodes for the nasopharynx. Rates of RLN metastases in patients with nasopharyngeal carcinoma is reported up to 94%. In constrast, the frequency of RLN metastases is relatively lower in the head and neck cancer from other sites (about 20%). The rate of RLN metastasis would be lower if the primary carcinoma didn't affect to posterior and lateral pharyngeal wall [4].

Retropharyngeal space is deep-seated and inaccessible to direct manual or endoscopic sampling. Open surgery in this region has a high risk of morbidity [5]. The management of clinically suspected retropharyngeal metastatic lesions without a malignant primary found is a challenge for physicians.

Recently, fine needle aspiration (FNA) with the guidance of ultrasound, CT or MRI was utilized to obtain histological diagnosis of retropharyngeal masses, which is an accurate and less invasive method, avoiding surgical biopsy and its possible morbidity [2, 3, 6]. Radiotherapy, as curative modality for metastatic carcinoma with an unknown primary, may be more suitable treatment for metastatic lesions in retropharyngeal space which is deep-seated, especially for radiation-sensitive carcinoma [7].

Here, we present results of a prospective study on radical radiotherapy for patients with retropharyngeal metastatic undifferentiated squamous cell carcinoma with an unknown primary, diagnosed with the help of transoral sonography-guided fine-needle aspiration.

## RESULTS

**Figure 1:**
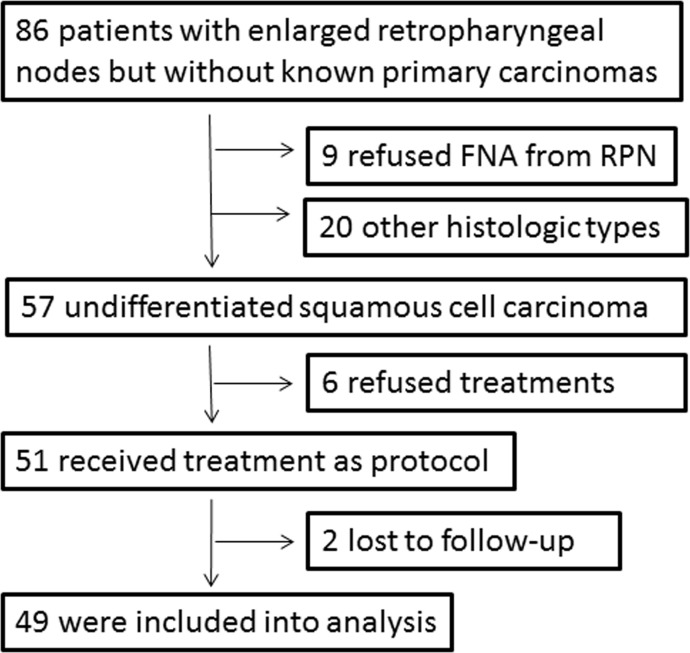
Flow diagram of the prospective study

Forty-nine patients, diagnosed as retropharyngeal metastatic undifferentiated squamous cell carcinoma, formed the basis of this report. Flow diagram of the prospective study is shown in Figure 1. The demographic data of enrolled patients and tumor characteristics are shown in Table 1. Combined treatment modalities and regimens of chemotherapy are listed in Table 2.

**Table 1:** Demographic and tumor characteristics

Characteristics	No. of patients (%)
Age	Median 47y (range, 18–72)
Gender	
Male	39 (80)
Female	10 (20)
Retrophayngeal masses	
Size	2.45cm (0.8-5.5cm)
Laterality	
Unilateral	32 (65)
Bilateral	17 (35)
Staging of neck disease	
N0	2 (4)
N1	14 (29)
N2a	3 (6)
N2b	6 (12)
N2c	16 (33)
N3	8 (16)

**Table 2:** Treatment modalities and regimens of chemotherapy

	No. of patients
Modalities	
Neo+con	15 (31)
Neo+adj	27 (55)
Regimens	
PF	26 (53)
TP	7 (14)
TPF	7 (14)

Neo+con: neoadjuvant chemotherapy followed by concurrent chemoradiotherapy; Neo+adj: neoadjuvant chemotherapy followed by RT alone plus adjuvant chemotherapy; PF: cisplatin and 5-fluorouracil; TP: docetaxel and cisplatin; TPF: docetaxel, cisplatin and fluorouracil.

### Control of retropharyngeal and cervical lymph node metastases

One patient experienced recurrence in retropharyngeal nodes and submaxillay nodes. One patient relapsed in submental nodes. The two failed patients both had the stage N3 nodes with diameter larger than 6 cm. Thus, retropharyngeal and cervical nodal disease was controlled in 96% of all patients.

### Appearance of occult primary cancer

During the follow-up, two patients experienced recurrence in the nasopharynx confirmed by biopsy. Two patients had relapses in the skull base diagnosed with serial PET-CT and MRI follow-ups. In the two patients who had relapses in the skull base, the lesions of the retropharyngeal nodes have had extracapsular extensions and affected surrounding skull base at diagnosis. After 22 and 36 months respectively, recurrences occurred in the skull base in the two patients. We regarded these two patients as patients having the primary in the nasopharynx because no primary cancer in other sites appeared and skull base relapse is a common failure pattern for NPC. No primary cancer other than of the nasopharynx was detected during the course of follow-up. Thus, the incidence of occult primary cancer appearance was 8%. One occult primary developed in a patient who also have nodes relapsed. All occult primaries in the nasopharynx developed in patients who were positive in Epstein-Barr virus serology (VCA-IgA). The mean time of appearance of occult primary following treatment was 37.25 months (22, 25, 36, 66 months respectively).

### Incidence of distant metastases and second primary cancers

Ten patients developed distant metastases. Bone metastasis was found in 8 patients, lung metastasis in 4 patients and liver metastasis in 3 patients. All the ten patients were free of failure in lymph nodes and primary carcinoma.

Second primary cancers developed in three patients, one in the pancreas, one in the kidney and another one in the rectal. All patients remained free of recurrences in the head and neck areas when diagnosed with the second primary cancers.

### The 5-year survival

**Figure 2:**
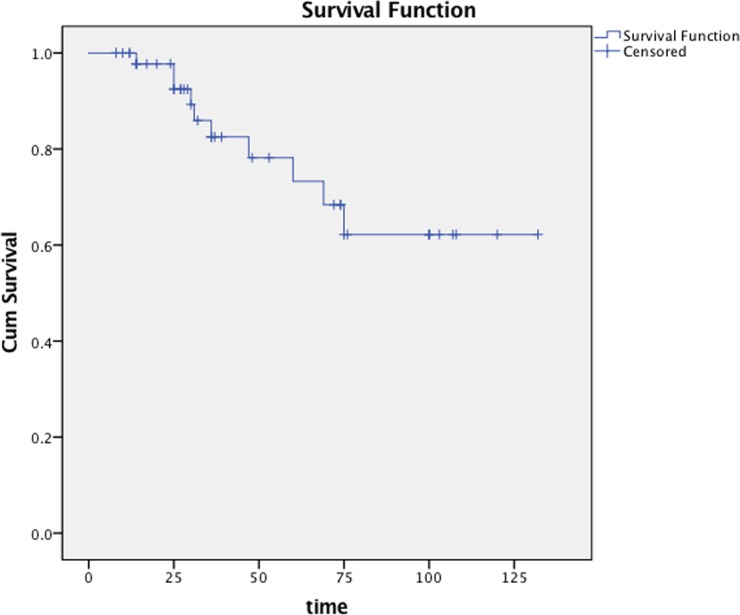
Overall survival of all patients

**Figure 3:**
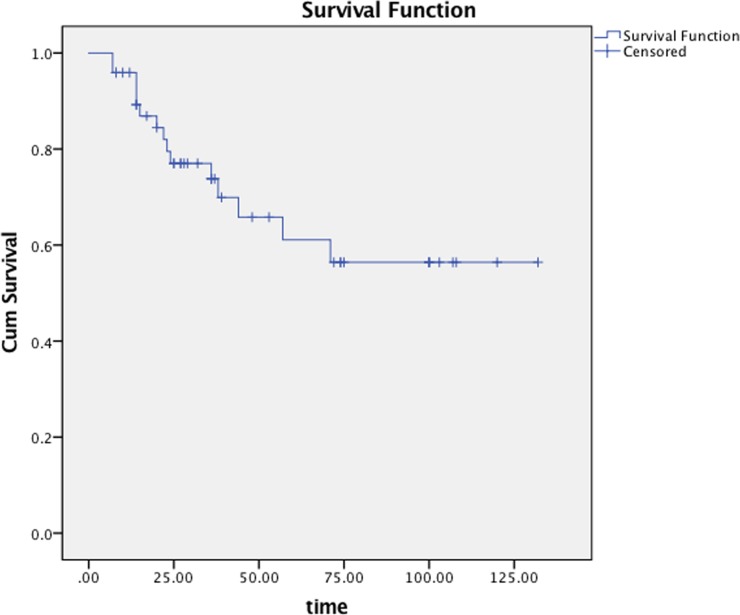
Progression-free survival of all patients

With a median follow-up of 37 months (range: 8-132 months), the 5-year overall survival (OS), progression-free survival (PFS), regional relapse free survival (RFS), distant metastasis free survival (DMFS) was 79.6% (Figure 2), 61.1% (Figure 3), 83.4%, 73.8%. Subgroup analyses showed that gender, bilateral cervical metastasis and N2-3 stage have a significant effect on PFS. Significant differences in the 5-year OS were observed between patients with N2-3 and those with N0-1 (60.9% vs. 92.9%, p = 0.043) (Table 3).

**Table 3:** Subset analysis on overall survival and progression free survival

	No. of patients	OS	P value	PFS	P value
Age (years)					
<=50	27	78%		63.6%	
>50	22	64.1%	0.32	56.7%	0.485
N stage					
N0-1	16	92.9%		85.7%	
N2-3	33	60.5%	0.043	43.9%	0.034
Chemo. Regimens					
PF	26	81.1%		67.5%	
TP/TPF	14	48.5%	0.297	48.8%	0.228
Modalities					
Neo+con	15	65.5%		62.8%	
Neo+adj	27	75.8%	0.827	63.4%	0.823

OS: overall survival; PFS: progression free survival; RT: radiotherapy; Neo+con: neoadjuvant chemotherapy followed by concurrent chemoradiotherapy; Neo+adj: neoadjuvant chemotherapy followed by RT alone plus adjuvant chemotherapy; PF: cisplatin and 5-fluorouracil; TP: docetaxel and cisplatin; TPF: docetaxel, cisplatin and fluorouracil.

### Toxicities

Grade 3/4 acute toxicities induced by chemotherapy included leukopenia (23%) and vomiting (8%). Nine (18%) patients experience grade 3 radiotherapy-related mucositis, and no grade 4 acute mucositis was observed. Common late adverse effects included xerostomia (57%) and hearing impairment (35%). The details of major late toxicities are summarized Table 4.

**Table 4:** The rates of major late toxicities

Toxicity	Grade 0 n (%)	Grade 1 n (%)	Grade 2 n (%)	Grade 3 n (%)	Grade 4 n (%)
Xerostomia	21(43)	21(43)	7(14)	0	0
Hearing impairment	32(65)	16(33)	1(2)	0	0
Temporal necrosis	49(100)	0(0)	0	0	0
Trismus	48(98)	1(2)	0	0	0
Neck fibrosis	31(63)	18(37)	0	0	0
Dysphagia	49(100)	0	0	0	0

## DISCUSSION

Retropharyngeal metastatic nodes from an unknown primary site in the head and neck were rarely reported. Retropharyngeal space is deep-seated and surrounded by vital vessels and nerves. Thus, the potential benefit of diagnosing and treating the lesions in this space should be weighed against the potential complications. The management of clinically suspected retropharyngeal metastatic lesions without a malignant primary is a challenge for physicians.

Debate still exists in the selection of treatment options for lymph nodes metastasis from the occult primary in the head and neck region [11–13]. The treatment options including surgery alone, surgery combined with radiotherapy to the involved neck, and primary irradiation to both sides of the neck plus the mucosal sites at risk had all been advocated. This controversy could be explained by lymph nodes metastasis from the occult primary in the head and neck region is a disease entity with heterogeneity. The factors influencing the choice vary with types of histology, positions of metastatic nodes, operability of the disease and the prevalence of peculiar subtypes of head and neck cancer.

Retropharyngeal metastatic undifferentiated squamous cell carcinoma from an unknown primary site could be considered as a particular type, especially in the NPC endemic area [14, 15]. Retropharyngeal lymph nodes are usually considered as the first-echelon lymph nodes for the nasopharynx. Rates of RLN metastases in patients with nasopharyngeal carcinoma vary between 63.6% and 94% [16–19] in the NPC endemic area. Wang et al [20] analyzed the lymph nodes metastatic patterns of 3100 patients with NPC and found 81.7% of them had retropharyngeal nodes metastasis. Tong et al [21] reported thirteen patients with metastatic nodes with undifferentiated squamous carcinoma from unknown origin and found that undifferentiated carcinoma was associated with better locoregional control and disease specific survival than squamous cell carcinoma. It's indicated that retropharyngeal metastatic undifferentiated squamous cell carcinoma possessed intrinsic radiosensitivity and the nasopharynx was the most potential original site.

Compared with NPC, the frequency of RLN metastases is lower in the non-nasopharyngeal head and neck cancer [4]. Dirix et al [22] reported a rate of RPLN metastases of 16% in a series of 208 patients with oropharyngeal cancer after evaluation of pretreatment CT scans. Shimizu et al [23] reported a rate of 13% of RPLN metastases for oropharyngeal cancers, with 29% for patients with posterior and lateral pharyngeal wall cancers and 0% for others sites. Yoshimoto et al [24] reported 2 patients had RPLN metastases at the time of diagnosis and 2 more developed recurrent cancer in the RPLN in 84 patients with base of tongue cancer, and three of these 4 patients had a tumor extending to the lateral pharyngeal wall. Although it's reported that unknown primaries are most likely to occur in the tonsillar area and base of tongue and tonsils, the rate of RPLN metastases is relatively lower for base of tongue cancer and tonsils cancer, especially without the affecting of posterior and lateral pharyngeal wall.

As for the volume of radiation, a better control of neck and potential mucosal primary was achieved in patients with head and neck cancer of unknown primary who received radiotherapy to both sides of the neck and the mucosa of the entire pharyngeal axis [25–27]. However, conflicting data were reported by authors who favored treatment of the ipsilateral neck only, stating that a thorough workup to discover the primary tumor was more worthwhile than prophylactic extensive irradiation because of the high morbidity such as xerostomia [28, 29]. As far as we known, the current prospective study was the largest study evaluating the treatment efficacy of retropharyngeal metastatic undifferentiated squamous cell carcinoma. To cover enough potential mucosal while reducing the radiation morbidity, prophylactic irradiation of nasopharyngeal mucosa as well as on both sides of the neck was used for patients in our study. Retropharyngeal and cervical nodal disease was controlled in 96% of all patients. The incidence of occult primary cancer appearance was 8%. No primary cancer other than of the nasopharynx was detected during the course of follow-up. All occult primaries in the nasopharynx developed in patients who were positive in Epstein-Barr virus serology (VCA-IgA). It's reflected that the radiation volume including the nasopharyngeal mucosa and bilateral neck is enough to cover the potential risk site and effective in preventing the emergence of primary, especially for those VCA-IgA positive.

Transoral sonography-guided fine-needle aspiration was employed based on the following reasons. Firstly, despite recent technical advances imaging modalities including CT, MRI and PET-CT (expensive and not accessible in many places), a tissue diagnosis remains a standard requirement to establish a diagnosis and to guide treatment. Secondly, retropharyngeal metastatic nodes are usually relatively smaller in size than cervical metastatic nodes, the diagnoses of retropharyngeal nodes just based on radiological imaging studies are always ambiguous, especially in those nodes absent of special characteristic (capsular enhancement or infiltration et al). Thirdly, for the patients presenting enlarged retropharyngeal nodes without neck adenopathy, transoral sonography-guided fine-needle aspiration is the most effective and minimally invasive alternative to obtain histologic diagnosis. The last but not the least, we considered the recruited patients as occult nasopharyngeal carcinoma based on typical type of histology, unique lymph node metastasis patterns and the epidemiology in the NPC endemic area. The FNA from retropharyngeal lymph nodes (the first-echelon lymph nodes for the nasopharynx and the closest area beside the nasopharynx) would be more important to diagnose and treat patients as occult nasopharyngeal carcinoma than FNA from neck nodes. All patients in current study acquired histology diagnoses with no adverse reaction during aspiration except the pain, which is minimal. No post procedural complications occurred. It's shown that transoral sonography-guided fine-needle aspiration is safe and accurate in obtaining the tissue diagnoses of lesions in retropharyngeal space.

The metastatic rate of the patients in the study was high indicating that retropharyngeal metastatic undifferentiated squamous cell carcinoma is an aggressive disease with a high risk of early dissemination. Systemic therapy had been a key part in the treatment for head and neck squamous cell carcinoma, especially concurrent chemotherapy [30–32]. Unfortunately, the experience with systemic therapy in patients with head and neck carcinoma of unknown primary is limited. We administered chemotherapy using cisplatin-base regimen combined with radiotherapy for forty-two (42/49, 86%) patients in current study based on two points. Firstly, patients with stage N2-3 accounted for 67% in current study. Secondly, combined chemoradiotherapy has demonstrated better efficacy in localregionally advanced head and neck cancer than radiotherapy alone. N2-3 was a risk factor for OS and PFS in the univariate analysis, but not significant in the multivariate analysis, which can be explained by small number of patients. But it cannot be totally eliminated that the chemotherapy adapted in current study improved the survival of patients with N2-3 so that the survival difference between N0-1 and N2-3 become too small to be tested in multivariate analysis.

In conclusion, radical radiotherapy to both the nasopharynx and bilateral neck can achieve excellent outcome with mild toxicities for patients with retropharyngeal metastatic undifferentiated cervical squamous cell carcinoma (SCC) from an unknown primary site. Transoral sonography-guided fine-needle aspiration is safe and accurate in obtaining the tissue diagnoses of lesions in retropharyngeal space. Systemic therapy to reduce metastatic rate should be further explored.

## MATERIALS AND METHODS

From January 2005 to January 2015, patients presenting with enlarged retropharyngeal nodes with or without cervical lymph nodes were referred to our department. The initial evaluation and work-ups included a detailed history, physical examinations, Epstein-Barr virus serology (VCA-IgA) and a CT or MRI scan of the head and neck. Endoscopy was performed for evaluation of the head and neck mucosa, and random biopsies from the nasopharynx, oropharynx, and hypopharynx were obtained at the time of endoscopy. PET-CT is not mandatory in the workup for the patients in the study.

The inclusion criteria of the study were: patients proven with retropharyngeal metastatic undifferentiated squamous cell carcinoma from an unknown primary site with the help of transoral sonography-guided fine-needle aspiration. The exclusion criteria included: patients with retropharyngeal malignant disease of other types of histology, rather than metastatic undifferentiated squamous cell carcinoma; patients with distant metastasis; patients who refused transoral sonography-guided fine-needle aspiration for enlarged retropharyngeal nodes.

Patients without a primary malignancy underwent transoral sonography-guided fine-needle aspiration to confirm the nature of enlarged retropharyngeal nodes. When undifferentiated squamous cell carcinoma was confirmed by histological examination, radical radiotherapy was given as primary treatment. The volume of radiation included the mucosa of the nasopharynx and the bilateral neck, eliminating the oropharynx, the hypopharynx and larynx. This study was performed with the approval of Institutional Review Boards of Fudan University Shanghai Cancer Center, and patients signed informed consents before treatments.

### Procedure of transoral sonography-guided fine-needle aspiration

**Figure 4:**
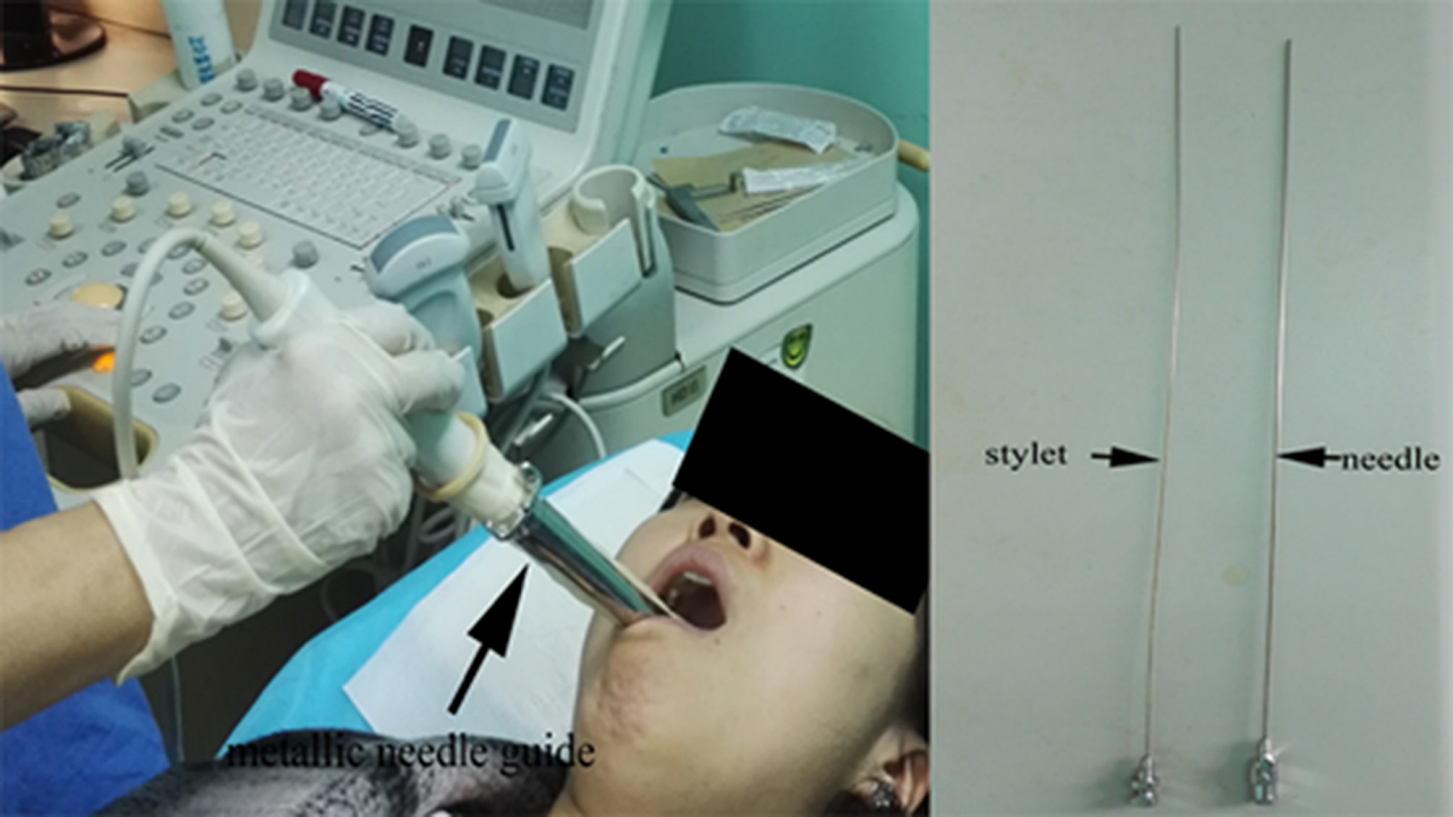
Photograph shows that endocavitary probe attached with a metallic needle guide has been inserted in oral cavity and the needle with stylet

Real-time ultrasound guidance was performed by YH, and subsequent biopsy was done by YZ or CD, with the patients in a supine position in an ultrasound suite. Local anesthesia was administered to the oropharyngeal mucosa (Figure 4). The endocavitary probe (Philips, IU-22) was used and was attached with a metallic needle guide. A 22cm 18-gauge needle was used to accommodate the length of the needle guide. A gray-scale sonogram was obtained to visualize the retropharyngeal masses on the monitor. Doppler ultrasound was used to identify the relation between the masses and the internal carotid artery (ICA) and internal jugular vein (IJV). A built-in biopsy guiding line was activated and cross the suspicious mass by adjusting the position of the probe. The needle with stylet was advanced into the mass through the guide. When the needle tip was within the limits of the target lesion, the stylet was pulled out. By applying moderate but continuous negative pressure using the hand holding the syringe and moving the needle to and fro, cellular material was aspirated from the retropharyngeal mass. After 20–30 seconds, aspiration was stopped and the syringe was slowly pulled out, and then the needle was pulled out. The cellular aspirate was recovered from the needle and smeared on two slides. The slides were sent to the cytopathology laboratory.

**Figure 5:**
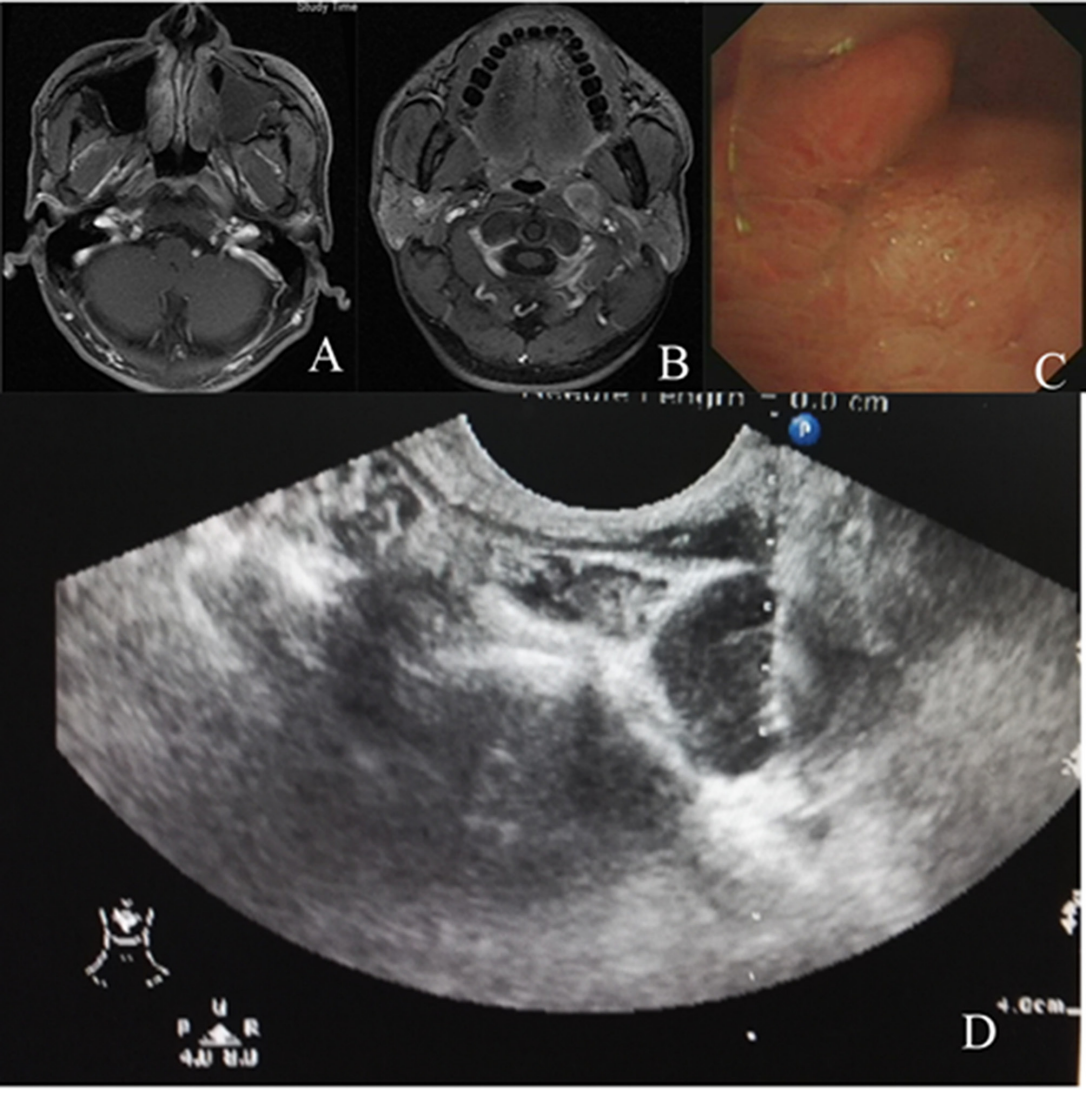
A male patient, 48y MRI **(A)** and nasopharyngo-fiberscope **(C)** revealed mucosa slightly thickening, but no evidence of malignancy was found after three times biopsy. Mass in the retropharyngeal space **(B)** was pathologically proven to be metastatic undifferentiated squamous carcinoma by transoral sonography-guided fine-needle aspiration **(D)**.

There was no adverse reaction during aspiration except the pain, which is minimal. No post procedural complications occurred (Figure 5)

### Treatment modalities

The intensity modulated radiotherapy (IMRT) technique was utilized for all patients. The target volumes were defined in accordance with the International Commission on Radiation Units and Measurements Reports 50 and 62. The gross tumor volume (GTV) included metastatic lymph nodes. The high-risk clinical target volume (CTV) should cover the entire nasopharynx, retropharyngeal and cervical lymphatics: levels II–V were outlined according to the recommendation by the Radiation Therapy Oncology Group (RTOG) consensus guidelines [8, 9]. The oropharynx, the hypopharynx and the larynx were not contoured as target volumes.

The metastatic nodes were irradiated with doses of 66 Gy or 70 Gy in 30 or 35 fractions. The radiation doses to CTV was 60 Gy in 30 or 35 fractions. The lower neck (below cricoid cartilage) was not irradiated if the ipsilateral neck (above cricoid cartilage) was not involved with metastatic nodes, 54 Gy in 30 or 35 fractions if the ipsilateral upper neck involved, and 60 Gy in 30 or 35 fractions if the ipsilateral lower neck involved.

Organs at risk were also delineated including the brain stem, spinal cord, temporal lobe, optic nerves, optic chiasm, lens, parotid glands, mandible, and temporomandibular joints. The normal tissue constraints and plan evaluation were in accordance with the Radiation Therapy Oncology Group 0225 protocol [10]. Inverse plans were optimized using the treatment planning system (Pinnacle version 7.6; Phillips, Milpitas, CA, USA).

If there was no contraindication, chemotherapy was administered for patients staged N2-3. The combination of induction chemotherapy followed by concurrent chemoradiotherapy was recommended. If concurrent chemotherapy cannot be tolerated or was refused by patients, induction plus adjuvant chemotherapy was combined with radiotherapy. Concurrent chemotherapy consisted of cisplatin (40 mg/m2 IV weekly or cisplatin 75 mg/m2 every 3 weeks) during radiation. The cisplatin-based regimens (cisplatin75 mg/m2 IV on day 1 plus docetaxel 75 mg/m2 IV on day 1 or 5-fu 500 mg/m2 d continuously IV on day1–5) were repeated every 3 weeks for 2–3 cycles for induction chemotherapy and every 4 weeks for 2 cycles for adjuvant phase.

### Assessment and follow-up

All patients were required to be followed up every 3 months in the first 2 years, every 6 months from the third year through the fifth year, and annually thereafter after the completion of their treatment. MRI or CT of head and neck, chest CT scan, and ultrasound of abdomen were performed 3 months after the completion of RT and every 6–12 months thereafter. Additional tests were ordered when indicated to evaluate local or distant relapse. For the patients who were suspected to have recurrence where it's impossible to get biopsy, PET-CT can be to help to determine the recurrence. The late adverse effects were recorded according to last follow-up status obtained by review of the patient record or telephone follow-up. Toxicities were graded according to the Radiation Morbidity Scoring Criteria of the Radiation Therapy Oncology Group.

### Statistics analysis

Endpoints for this study were metastatic nodes control, the appearance of primary tumor, overall survival and treatment-related toxicities. Survival data were analyzed by the Kaplan-Meier method. Log-rank test was used to test the statistical difference between various subgroups. A significance level of 5% (two-sided) was used for all tests. All analyses were performed in SPSS 13.0.
